# Real‐World Clinical Outcomes and Corticosteroid Tapering in Telitacicept‐Based Therapy for Ocular Myasthenia Gravis: An Exploratory Analysis by Prior Treatment History

**DOI:** 10.1002/brb3.71816

**Published:** 2026-10-08

**Authors:** Siyu Xie, Wenli Song, Ran Guo, Chunrong Li, Manman Zhang, Yifan Cheng, Wenting Guo, Bo Jin, Jia Li, Yiqi Wang

**Affiliations:** ^1^ The Second Clinical Medical College of Hangzhou Normal University Hangzhou China; ^2^ Center For Rehabilitation Medicine, Department of Neurology Zhejiang Provincial People's Hospital (Affiliated People's Hospital, Hangzhou Medical College) Hangzhou China; ^3^ Center For Rehabilitation Medicine, Department of Pain Management Zhejiang Provincial People's Hospital, Affiliated People's Hospital, Hangzhou Medical College Hangzhou PR China; ^4^ Department of Neurology School of Medicine Sir Run Run Shaw Hospital Zhejiang University Hangzhou China; ^5^ The First Affiliated Hospital of Wenzhou Medical University Wenzhou City China

**Keywords:** corticosteroid tapering, minimal symptom expression, naïve ocular myasthenia gravis, refractory ocular myasthenia gravis, telitacicept

## Abstract

**Introduction:**

Ocular myasthenia gravis (OMG) is an autoantibody‐mediated disease characterized by extraocular muscle weakness. Telitacicept has shown potential in treating autoimmune diseases, but its efficacy and corticosteroid‐tapering outcomes in OMG remain unexamined.

**Methods:**

This retrospective, multicenter study evaluated the efficacy and corticosteroid‐tapering outcomes of telitacicept in 19 OMG patients (11 with refractory OMG and eight with newly/reinitiated treatment OMG). The primary endpoint was the proportion of patients achieving minimal symptom expression (MSE) within 6 months. Secondary endpoints included changes in Myasthenia Gravis Activities of Daily Living (MG‐ADL) scores and reductions in daily prednisone dosage, cumulative response rate (CRR), and short‐term tolerability.

**Results:**

Following 6 months of telitacicept treatment, 68.42% of the overall cohort achieved MSE, with the median MG‐ADL score significantly decreasing from 3.00 to 0.00 (*p* < 0.0001). Daily prednisone dosage substantially decreased: the median daily prednisone dose fell from 20.00 to 5.00 mg (*p* < 0.0001), and 84.21% of patients received prednisone at ≤5 mg/day at month 6. All patients met the predefined initial response criterion within 5 weeks. The refractory and newly/reinitiated treatment groups achieved final MSE rates of 72.7% and 62.5%, respectively, and both groups showed significant reductions in prednisone dependence. This cohort had no serious adverse events.

**Conclusion:**

In this exploratory cohort, telitacicept‐based therapy may represent a promising option for OMG, with favorable clinical outcomes, substantial prednisone dose reduction, and acceptable short‐term tolerability in patients with different prior treatment histories. Further prospective controlled studies are warranted.

## Graphical abstract

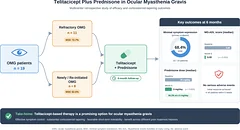

**Abbreviations**
AChR: acetylcholine receptor
ANOVA: analysis of variance
APRIL: A proliferation‐inducing ligand
BAFF: B‐cell activating factor
CRR: cumulative response rate
FcRn: neonatal Fc receptor
GMG: generalized myasthenia gravis
IQR: interquartile range
LMM: linear mixed model
MG: myasthenia gravis
MG‐ADL: Myasthenia Gravis Activities of Daily Living
MGFA: Myasthenia Gravis Foundation of America
MSE: minimal symptom expression
nOMG: newly/reinitiated treatment Ocular Myasthenia Gravis
NSIST: non‐steroidal immunosuppressive therapy
OMG: ocular myasthenia gravis
rOMG: refractory ocular myasthenia gravis
SD: standard deviation

## 1 Introduction

Myasthenia gravis (MG) is an autoantibody‐mediated disease in which pathogenic autoantibodies disrupt neuromuscular transmission (Fichtner et al. 2020). Ocular myasthenia gravis (OMG), classified as Myasthenia Gravis Foundation of America (MGFA) Class I and characterized by fluctuating ptosis and diplopia, accounts for approximately 15% of MG cases and significantly impairs quality of life (Gilhus 2016). Current management begins with acetylcholinesterase inhibitors, which show limited efficacy for diplopia. First‐line immunotherapy consists of oral glucocorticoids, which can rapidly restore ocular motility (Kupersmith et al. 1996). However, most OMG patients require prolonged prednisone for months or even years to maintain therapeutic efficacy (Verma et al. 2021), leading to metabolic, skeletal, and infectious complications (Evoli and Damato 2023; Menon et al. 2021). For patients with steroid intolerance, dependence, or inadequate response, non‐steroidal immunosuppressive therapies (NSISTs), including azathioprine, mycophenolate mofetil, tacrolimus, and cyclosporine, are commonly used as adjunctive or alternative agents (Omata et al. 2019). However, approximately 20% of patients develop refractory OMG with persistent symptoms.

Biologically targeted therapies, such as neonatal Fc receptor antagonists and complement C5 inhibitors, have demonstrated efficacy in randomized controlled trials for patients with generalized MG (GMG) (Howard et al. 2021; Marini et al. 2025, Jin et al. 2025). However, evidence specifically for OMG remains scarce. Telitacicept is a novel recombinant fusion protein that neutralizes B‐cell activating factor (BAFF) and a proliferation‐inducing ligand (APRIL), thereby inhibiting B lymphocyte maturation and plasma cell survival at multiple stages (Yao et al. 2021; Dhillon 2021; Vincent et al. 2013). Telitacicept was approved for systemic lupus erythematosus in 2021 (Dhillon 2021) and generalized MG in 2025 (Yin et al. 2024). A recent case series has suggested potential efficacy of telitacicept in refractory OMG (Lin et al. 2025), but systematic evaluation in OMG is lacking.

Given the established efficacy of telitacicept in GMG, this study aims to evaluate the clinical efficacy and concurrent corticosteroid reduction in OMG patients. We also compared therapeutic responses between newly/reinitiated treatment and refractory subgroups.

## 2 Materials and Methods

### 2.1 Study Design and Patients

This retrospective multicenter study (October 2022–June 2025) enrolled OMG patients from the Departments of Neurology at Zhejiang Provincial People's Hospital and the First Affiliated Hospital of Wenzhou Medical University. Telitacicept was administered to all enrolled patients as either an add‐on or an initial therapy, with regular follow‐up for at least 6 months. Inclusion criteria were as follows: (1) patients diagnosed with MG according to the Chinese Guidelines for the Diagnosis and Treatment of Myasthenia Gravis (2025 edition) (Chinese Society of Neuroimmunology 2025) by two experienced neurologists; (2) patients classified as MGFA Class I, with ptosis and/or diplopia and no documented history of generalized MG, MG‐Activities of Daily Living (ADL) score ≥2 at the first visit; (3) with no biologics treatment within previous 3 months. (4) Previous immunotherapy regimens remained unchanged before telitacicept treatment: corticosteroids at a stable dose for at least 1 month and NSISTs (tacrolimus or azathioprine) at the same dose for at least 3 months. The study protocol was approved by the Institutional Ethics Committee of Zhejiang Provincial People's Hospital and conducted in accordance with the Declaration of Helsinki.

Patients were classified into refractory OMG (rOMG) and newly/reinitiated treatment OMG (nOMG) groups according to prior treatment history. We defined rOMG as persistent ocular symptoms despite stable background immunotherapy. The nOMG group was operationally defined as comprising patients who were either newly diagnosed with ocular‐onset MG and had never received MG‐directed treatment or had not received no MG‐directed treatment for at least 2 years before enrollment. Telitacicept was added to ongoing background immunotherapy in the rOMG group, whereas the nOMG group received oral prednisone and telitacicept concurrently.

### 2.2 Assessments

Initially, patients received telitacicept at 160  or 240 mg once weekly (QW), or 160 mg every 2 weeks (Q2W). Based on periodic clinical evaluations and individualized therapeutic responses, we progressively extended or adjusted dosing intervals and regimens during follow‐up, while gradually reducing concurrent prednisone doses. Detailed patient‐specific telitacicept dosing trajectories and interval adjustments across the 6 months are provided in Table S1.

We retrospectively collected basic clinical data, including sex, age at MG onset, clinical presentation, disease duration, MG antibody status, thymic status, history of thymectomy, baseline prednisone dose, and baseline NSIST use. For patients with a history of thymectomy, the timing of surgery relative to telitacicept initiation was extracted from medical records. Notably, all thymectomies were performed at least 5 years before telitacicept initiation (Tables 2 and 3).

All patients were followed up for at least 6 months. Clinical outcomes were assessed at weeks 1, 2, 3, and 4, and at months 2, 3, 4, 5, and 6 post‐treatment initiation. At each follow‐up visit, we recorded MG‐ADL score, daily prednisone dosage, and adverse events. Minimal symptom expression (MSE) was defined as an MG‐ADL score of 0 or 1 (Fang et al. 2025). Cumulative response rate (CRR) was defined as the proportion of patients who had achieved a ≥1‐point reduction in the MG‐ADL score from baseline at or before each assessment. If a patient maintains MSE for a certain period, we define this period as the duration of sustained MSE. The duration of sustained MSE was calculated as the total continuous period during which patients remained in MSE status without relapse.

### 2.3 Endpoints

The primary efficacy endpoint was the proportion of patients achieving MSE within 6 months. Secondary efficacy endpoints assessed at each time point from baseline to month 6 included (1) change in MG‐ADL score, (2) reduction in mean daily prednisone dosage, (3) CRR at each observation time point, (4) proportion of patients achieving a daily prednisone dose≤5 mg at month 6, and (5) duration of sustained MSE.

### 2.4 Adverse Events and Short‐Term Tolerability

We recorded and described all adverse events occurring during the treatment period 2 using the Common Terminology Criteria for Adverse Events, version 5.0.

### 2.5 Statistical Analysis

Statistical analyses were performed using SPSS 25.0 and GraphPad Prism 10.1.2. Descriptive statistics summarized baseline characteristics; continuous variables were presented as mean ± standard deviation (SD) or median (IQR) based on distribution (Shapiro–Wilk test), and categorical variables were presented as *n* (%) with chi‐square or Fisher's exact tests. Group comparisons of continuous variables used the Mann–Whitney U test. The proportion of patients achieving MSE was analyzed using Kaplan–Meier curves. Longitudinal changes were assessed using repeated‐measures analysis of variance (ANOVA) (normal distribution) or the Friedman test (non‐parametric). Linear mixed models compared MG‐ADL and prednisone trajectories between groups, with time as a continuous fixed effect, group as a categorical fixed effect, their interaction, and patient‐specific random intercepts. All tests were two‐sided, with significance set at *p* < 0.05.

## 3 Result

### 3.1 Baseline Characteristics of Patients

We included 19 patients with OMG who received telitacicept treatment for at least 6 months across two centers. The two retrospective cohorts comprised 11 patients with rOMG (57.89%) and eight patients with nOMG (42.11%). Baseline clinical characteristics were comparable between subgroups (Table 1).

**TABLE 1:** Baseline characteristics of enrolled patients treated with telitacicept.

Clinical parameters	Total (*n* = 19)	Refractory OMG (*n* = 11)	Newly/reinitiated treatment OMG (*n* = 8)	*p* value
Gender, *n* (%)				1.000
Male	11 (57.89)	6 (54.55)	5 (62.50)	
Female	8 (42.11)	5 (45.45)	3 (37.50)	
Disease duration (months) (IQR)	26.13 (6.98–131.53)	60.00 (18.00–131.53)	6.98 (0.52–94.45)	0.341
Onset age (years) (mean ± SD)	33.89 ± 24.78	32.45 ± 24.99	35.88 ± 26.06	0.776
Symptom, *n* (%)				0.170
Ptosis	9 (47.37)	7 (63.64)	2 (25.00)	
Ptosis and diplopia	10 (52.63)	4 (36.36)	6 (75.00)	
Thymus status, *n* (%)				1.000
Normal	13 (68.42)	7 (63.64)	6 (75.00)	
Thymoma	3 (15.79)	2 (18.18)	1 (12.50)	
Thymic hyperplasia	3 (15.79)	2 (18.18)	1 (12.50)	
Thymectomy, *n* (%)				1.000
No	16 (84.21)	9 (81.82)	7 (87.50)	
Yes	3 (15.79)	2 (18.18)	1 (12.50)	
Antibody, *n* (%)				0.603
Negative	5 (26.32)	2 (18.18)	3 (37.50)	
AChR‐Ab	14 (73.68)	9 (81.82)	5 (62.50)	
Baseline MG‐ADL score (IQR)	3.00 (3.00‐6.00)	3.00 (3.00‐4.50)	5.50 (4.50‐6.00)	0.085
Initial dose of Pri (mg/day) (IQR)	20.00 (15.00‐35.00)	30.00 (17.50‐50.00)	17.50 (15.00‐22.50)	0.335

*Note*: Total MG‐ADL scores range from 0 (normal) to 24 (severe).

Abbreviations: Ab, antibody; AChR‐Ab, acetylcholine receptor antibodies; IQR, interquartile range; MG‐ADL, Myasthenia Gravis–Activities of Daily Living; OMG, ocular myasthenia gravis; Pri, prednisone; SD, standard deviation.

In the cohort (*n* = 19), the male‐to‐female ratio was 11:8. The mean onset age was 33.89 ± 24.78 years. The median disease duration for the entire cohort was 26.13 months (IQR 6.98–131.53). All patients presented with ptosis, including 10 (52.63%) with comorbid diplopia. Thymomas or thymic hyperplasia were identified in three patients (15.79%), three of whom underwent thymectomy, with the remaining cases exhibiting normal thymic status. Serological testing revealed that 14 patients (73.68%) were positive for acetylcholine receptor (AChR) antibodies, and five patients (26.32%) were antibody‐negative.

### 3.2 Overall Treatment Response in Patients Receiving Telitacicept‐Based Regimens

In the 19 patients with OMG treated with telitacicept, marked clinical improvement and a significant corticosteroid‐tapering outcomes effect were observed after 6 months of treatment. As shown in the Kaplan–Meier curve, the proportion of patients achieving MSE increased progressively over time: seven patients (36.84%) reached MSE within 2 months, nine patients (47.36%) reached MSE within 3 months, 11 patients (57.89%) reached MSE within 4 months, and 13 patients (68.42%) reached MSE at the end of observation. The remaining six patients (three rOMG and three nOMG) showed obvious clinical improvement but still experienced mild symptoms at the last follow‐up (Figure 1A). No participant had uncontrolled MG symptoms. Among the 13 patients who achieved MSE, the median duration of sustained MSE was 3.00 (IQR 2.00–4.00) months, ranging from 0.25 to 6 months.

**FIGURE 1:**
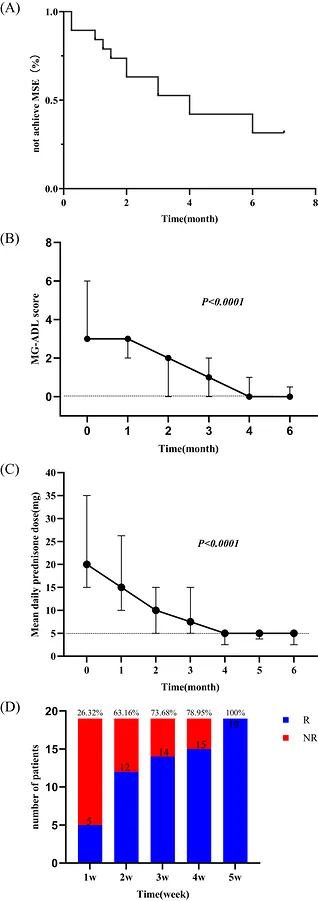
Clinical outcomes following telitacicept treatment in OMG. (A) Kaplan–Meier survival curve illustrating the probability of not achieving MSE over 6 months. (B) There was a significant decline in median MG‐ADL scores during 6 months (*p* < 0.0001). (C) A reduction in median daily prednisone dose was observed in 19 patients (*p* < 0.0001). (D) Cumulative response rate (CRR) over time with telitacicept treatment. MSE was defined as an MG‐ADL score of 0 or 1. CRR was defined as the proportion of patients who had achieved a ≥1‐point reduction in the MG‐ADL score from baseline at or before each assessment.

MG‐ADL scores improved rapidly and sustainably (Figure 1B). The median MG ‐ADL score decreased from 3.00 (IQR 3.00–6.00) at baseline to 2.00 (IQR 0.00–2.00) at month 2, and further to 0.00 (IQR 0.00–0.50) at month 6 (Friedman's test, *p* < 0.0001). Post hoc analysis confirmed significant reductions from baseline at months 2, 3, 4, and 6 (all *p* < 0.001).

A total of 18 patients received prednisone before telitacicept initiation. The median daily dose of prednisone decreased from 20.00 mg (IQR 15.00–35.00) at baseline to 10.00 mg (IQR 5.00–15.00) at month 2, 7.50 mg (IQR 5.00–15.00) at month 3, and finally 5.00 mg (IQR 2.50–5.00) at month 6, demonstrating a significant reduction in the mean daily prednisone dosage over the study period (Friedman's test, *p* < 0.0001) (Figure 1C), reflecting substantial daily prednisone dose reduction during telitacicept‐based therapy. At month 6, 16 patients (84.21%) were receiving prednisone at a dose of ≤5 mg/day, a dose considered acceptable for long‐term maintenance in MG.

CRR increased rapidly after telitacicept treatment initiation (Figure 1D). At week 1, five patients (26.32%) showed an initial response (≥1‐point MG‐ADL reduction). CRR reached 63.16% (12 patients) by week 2, 73.68% at week 3, and 78.95% at week 4. By week 5, all 19 patients (100%) had responded, reflecting an early onset pattern of symptom improvement in these patients.

Throughout the 6‐month follow‐up period, no serious adverse events or events leading to treatment discontinuation were observed. The only recorded adverse events were mild injection‐site reactions (erythema and transient localized pain), which occurred in three patients (15.79%) during the first month of therapy. All events were Grade 1 per CTCAE v5.0 and resolved spontaneously without dose interruption or specific medical management. Detailed safety event profiles are summarized in Table S2.

### 3.3 Treatment Response in rOMG Patients

Eleven patients with rOMG were included (five females and six males). The mean age at disease onset was 32.45 ± 24.99 years, with a median disease duration of 60.00 months (IQR 18.00–131.53). Two had childhood‐onset disease (onset age <18 years). Baseline symptoms included ptosis (11/11) and diplopia (4/11). Serological testing revealed AChR antibodies positive in nine patients (81.82%); two patients (18.18%) were antibody‐negative. Two patients (18.18%) were diagnosed with thymoma, and two patients (18.18%) had thymic hyperplasia, two of whom underwent thymectomy (Table 2).

**TABLE 2:** Baseline characteristics of refractory patients treated with telitacicept.

Patient No.	1	2	3	4	5	6	7	8	9	10	11
Onset age (years)	42	9	25	66	17	4	74	64	16	26	14
Gender	Male	Male	Female	Male	Male	Female	Male	Male	Female	Female	Female
Disease duration (months)	26.13	408	192	60	12	60	24	2.3	170.47	92.6	12
OMG duration (months)	26.13	408	192	60	12	60	24	2.3	170.47	80.0^a^	12
MGFA class	I	I	I	I	I	I	I	I	I	I	I
Symptom	Ptosis	Ptosis	Ptosis and diplopia	Ptosis and diplopia	Ptosis	Ptosis	Ptosis	Ptosis and diplopia	Ptosis and diplopia	Ptosis	Ptosis
Thymus status	Normal	Thymoma	Normal	Thymoma	Normal	Normal	Normal	Normal	Thymic hyperplasia	Thymic hyperplasia	Normal
Thymectomy	N	Y	N	N	N	N	N	N	N	Y	N
Timing of thymectomy	N	Unspecified^b^	N	N	N	N	N	N	N	2017	N
Antibody	AChR‐Ab (+)	Negative	AChR‐Ab (+)	AChR‐Ab (+)	Negative	AChR‐Ab (+)	AChR‐Ab (+)	AChR‐Ab (+)	AChR‐Ab (+)	AChR‐Ab (+)	AChR‐Ab (+)
Baseline MG‐ADL score	2	3	6	6	3	3	3	3	6	2	3
Initial dose of Pri (mg/day)	20	30	60	60	60	20	30	12.5	15	0	40
Previous MG medications	AChEIs, Pri	Pri, TAC 1 mg qd,	AChEIs, Pri, TAC 1.5 mg qd	AChEIs, Pri, TAC 1.5 mg bid	AChEIs, Pri	AChEIs, Pri	Pri	Pri, AZA, 50 mg bid	AChEIs, Pri, AZA 50 mg bid	TAC 1 mg bid	Pri, AZA 50 mg bid
Basic treatment pre‐telitacicept	Pri	Pri	Pri	AChEIs, Pri, TAC	AChEIs, Pri	AChEIs, Pri	Pri, TAC	Pri, AZA	Pri	TAC	Pri, AZA
Dosage of telitacicept	160 mg/week	160 mg/week	160 mg/week	240 mg/week	240 mg/week	160 mg/week	240 mg/week	160 mg/2 week	160 mg/week	160 mg/week	160 mg/2week
Time to respond (weeks)	1	2	2	4	3	2	2	5	1	5	2
MG‐ADL score at month 6	0	1	0	1	0	2	2	0	3	0	0

*Note*: Total MG‐ADL scores range from 0 (normal) to 24 (severe).

Abbreviations: Ab, antibody; AChEI, acetylcholinesterase inhibitor; AChR‐Ab, acetylcholine receptor antibodies; AZA, azathioprine; MG‐ADL, Myasthenia Gravis–Activities of Daily Living; MGFA, Myasthenia Gravis Foundation of America; N, no; OMG, ocular myasthenia gravis; Pri, prednisone; TAC, tacrolimus; Time to response, defined as a ≥1‐point reduction in MG‐ADL score; Y, yes.

^a^For patient 10, the total disease duration is 92.6 months; however, this patient developed transient generalized symptoms for approximately 1 year following thymectomy in 2017 and subsequently reverted to purely ocular involvement. Therefore, the duration of pure OMG is estimated as ∼80 months (92.6−12.6 months). For all other patients, OMG duration equals total disease duration.

^b^ Patient 2 underwent thymectomy >10 years before telitacicept initiation (exact calendar year was unspecified in the medical record).

Prior immunotherapy included prednisone in 10 patients (90.91%), tacrolimus in three patients (27.27%), and azathioprine in two patients (18.18%). Throughout the follow‐up, all patients continued telitacicept treatment. Tacrolimus was discontinued in two patients (at months 1 and 2), and prednisone was tapered in all patients (Table 2).

The proportion of patients achieving MSE in the rOMG group increased over time (Figure 2A). Three patients (27.27%) reached MSE by month 2, 5 patients (45.45%) by month 3, 7 patients (63.64%) by month 4, and 8 patients (72.73%) achieved MSE at the end of observation. The median duration of sustained MSE was 3.00 (IQR 2.00–4.00) months; individual durations were 6, 4, 4, 2, 3, 0.25, 3, and 2 months, respectively, suggesting sustained symptom improvement in these patients.

**FIGURE 2:**
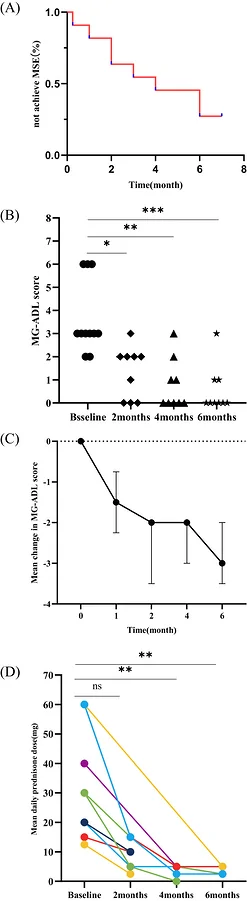
Clinical outcomes following telitacicept treatment in refractory OMG. (A) Kaplan–Meier survival curve illustrating the probability of not achieving MSE over 6 months. (B, C) Compared with baseline, the MG‐ADL score decreased significantly from 2 months to the end of the study (*p* < 0.05). (D) The median daily prednisone dose significantly decreased from 4 months to 6 months compared with baseline (*p* < 0.05). In panels B and C, different symbols (circles, diamonds, triangles, asterisks) represent individual patient data points at each time point; they are used for visual distinction only and do not denote statistical significance. MSE was defined as an MG‐ADL score of 0 or 1.

MG‐ADL scores improved significantly (Figure 2B). The median MG‐ADL score decreased significantly from 3.00 (IQR 3.00–4.50) at baseline to 1.00 (IQR 0.00–2.00) at month 2 and further to 0.00 (IQR 0.00–0.75) at month 4 and 0.00 (IQR 0.00–0.25) at month 6 (Friedman's test, *p* = 0.0001). As shown in Figure 2C, all patients (100%) achieved at least a two‐point reduction in MG‐ADL scores by month 2, with a median reduction of 3.00 (IQR 2.00–3.50) by month 6. The CRR increased progressively from 18.18% at week 1 to 63.64% at week 2, 72.73% at week 3, and 81.82% at week 4, reaching 100% by week 5.

A substantial daily prednisone dose reduction was also observed in the rOMG group (Figure 2D). Accordingly, the median daily dose of prednisone decreased from 30.00 mg (IQR 17.50–50.00) at baseline to 5.00 mg (IQR 1.25–5.00) at 4 months, and further reduced to a median of 2.50 mg (IQR 2.50–5.00) at 6 months (Friedman's test, *p* = 0.0023). Notably, one patient (P10) did not receive any prednisone during the study period.

### 3.4 Treatment Response in nOMG Patients

Eight patients with nOMG were included (five males, three females). The mean age at disease onset was 35.88 ± 26.06 years (range 3‐67 years), and two patients had childhood‐onset disease (onset age <18 years). The median disease duration before treatment was 6.98 months (IQR 0.52–94.45). Three patients (Patients 1, 4, 6) had received prior therapy (acupuncture, AChEIs, and prednisone) 2 years before enrollment but had been off treatment for >2 years. The remaining five patients had no prior MG treatment. All eight patients (100%) presented with ptosis, including six (75.0%) with comorbid diplopia. Serological testing showed AChR antibody positivity in five patients (62.5%), and three patients (37.5%) were antibody‐negative. One patient had thymoma (treated through surgery), and one had thymic hyperplasia (Table 3).

**TABLE 3:** Baseline characteristics of newly/reinitiated treatment patients treated with telitacicept.

Patient No.	1	2	3	4	5	6	7	8
Onset age (years)	4	67	58	3	52	19	25	59
Gender	Male	Female	Male	Male	Female	Male	Male	Female
Disease duration (months)	256.3	0.27	0.23	460.1	0.6	40.5	1.97	12
OMG duration (months)	256.3	0.27	0.23	460.1	0.6	40.5	1.97	12
MGFA class	I	I	I	I	I	I	I	I
Symptom	Ptosis and diplopia	Ptosis	Ptosis	Ptosis and diplopia	Ptosis and diplopia	Ptosis and diplopia	Ptosis and diplopia	Ptosis and diplopia
Thymus status	Thymic hyperplasia	Normal	Thymoma	Normal	Normal	Normal	Normal	Normal
Thymectomy	N	N	Y	N	N	N	N	N
Timing of thymectomy	N	N	2017	N	N	N	N	N
Antibody	AChR‐Ab (+)	AChR‐Ab (+)	AChR‐Ab (+)	AChR‐Ab (+)	Negative	AChR‐Ab (+)	Negative	Negative
Baseline MG‐ADL score	5	3	3	6	6	6	6	6
Initial dose of Pri (mg/d)	15	15	20	10	60	20	15	30
Previous MG medications	Ocular block needle	N	N	AChEIs	N	Pri	N	N
Combined treatment with telitacicept	Pri	Pri	Pri	Pri	Pri	Pri	Pri	Pri
Dosage of telitacicept	160 mg/week	160 mg/week	160 mg/week	160 mg/week	240 mg/week	160 mg/week	160 mg/week	240 mg/week
Time to respond (weeks)	1	5	5	1	2	1	2	3
MG‐ADL score at month 6	0	0	0	2	0	0	2	2

*Note*: Total MG‐ADL scores range from 0 (normal) to 24 (severe).

Abbreviations: AChEI, acetylcholinesterase inhibitor; AChR‐Ab, acetylcholine receptor antibodies; Ab, antibody; MG‐ADL, Myasthenia Gravis–Activities of Daily Living; MGFA, Myasthenia Gravis Foundation of America; N, no; OMG, ocular myasthenia gravis; Pri, prednisone; Time to response, defined as a ≥1‐point reduction in MG‐ADL score; Y, yes.

All patients underwent a clinical follow‐up period of 6 months (Figure 3A). During this period, 3 patients (37.50%) reached MSE by month 2, four patients (50.00%) by month 3, and five patients (62.50%) by month 4 and month 6. The median duration of sustained MSE was 4.00 (IQR 2.50–5.00) months; individual durations were 6, 2, 4, 3, and 4 months, respectively. The remaining three patients did not achieve MSE, with symptoms limited to mild diplopia without impact on daily living.

**FIGURE 3:**
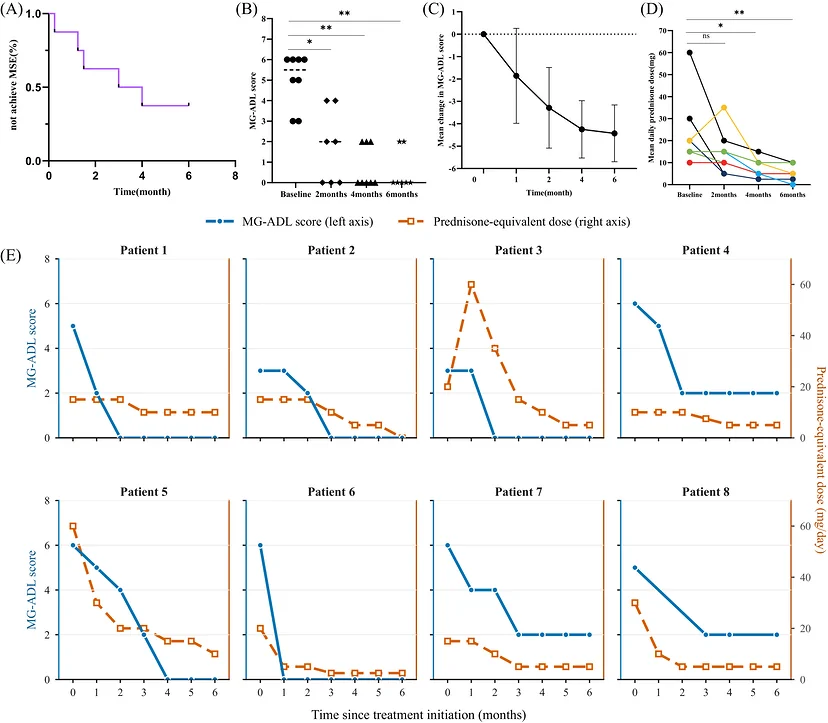
Clinical outcomes following telitacicept treatment in newly/reinitiated treatment OMG. (A) Kaplan–Meier survival curve illustrating the probability of not achieving MSE over 6 months. (B, C) Compared with the baseline, the MG‐ADL score showed a significant decrease from 2 months to the end of the study (*p* < 0.05). (D) The median daily prednisone dose was significantly reduced from 4 months to 6 months compared with the baseline (*p* < 0.05). (E) Individual longitudinal trajectories of MG‐ADL scores and daily prednisone doses in each of the eight patients. In panels B and C, different symbols (circles, diamonds, triangles, asterisks) represent individual patient data points at each time point; they are used for visual distinction only and do not denote statistical significance. MSE was defined as an MG‐ADL score of 0 or 1.

Eight nOMG patients were analyzed. As observed in the overall population, MG‐ADL scores declined markedly following telitacicept treatment (Figure 3B,C). The median MG‐ADL score decreased significantly from 5.50 (IQR 4.50–6.00) at baseline to 2.00 (IQR 0.00–3.00) at month 2, and further to 0.00 (IQR 0.00–1.00) at month 6 (Friedman's test, *p* < 0.001). The MG‐ADL score dropped by up to 4 points at month 1, and by month 2, all patients (100%) had achieved at least a 1.5‐point reduction. The CRR increased progressively from 37.50% at week 1 to 62.50% at week 2, 75.00% at week 3, reaching 100% by week 5, demonstrating a rapid onset of clinical improvement.

Correspondingly, in this cohort (Figure 3D), all eight patients received telitacicept in combination with prednisone. Six patients (75.0%) had an initial daily prednisone dose of ≤20 mg, and two patients received a daily prednisone dose of >30 mg at baseline. There was a significant reduction in the median daily prednisone dose from 17.50 mg (IQR 15.00–22.50) at baseline to 12.50 mg (IQR 8.75–16.25) at month 2, and further to 5.00 mg (IQR 5.00‐8.75) at 4 months, which was sustained at 6 months, with a median reduction of 71.43% compared to baseline (Friedman's test, *p* = 0.0002).

To further illustrate the temporal relationship between clinical improvement and corticosteroid tapering in individual patients, we plotted the longitudinal trajectories of MG‐ADL scores overlaid with daily prednisone doses for each of the eight patients in the newly/reinitiated cohort (Figure 3E), suggesting that the combination of telitacicept and low‐dose prednisone was associated with marked efficacy.

### 3.5 Exploratory Subgroup Analyses by Prior Treatment History

To further explore the potential impact of prior treatment history on clinical responses to telitacicept, an exploratory comparison of treatment trajectories was conducted between the rOMG and nOMG groups.

#### 3.5.1 MG‐ADL Score Trajectories

Linear mixed model (LMM) was used to compare the longitudinal changes in MG‐ADL scores between the rOMG (*n* = 11) and nOMG (*n* = 8) groups. MG‐ADL scores decreased significantly over time, with estimated mean monthly reductions of 0.56 points in the nOMG group and 0.37 points in the rOMG group. No statistically significant difference in the rate of improvement was detected between the groups (time × group interaction *β* = 0.19, 95% CI: −0.07 to 0.44, *p* = 0.144). These findings were consistent with the non‐parametric analyses (Friedman test, *p* < 0.001 for both groups), supporting the robustness of the observed improvement.

Complementing this overall trajectory analysis, the CRR offered additional insight into the timing of the initial response. As shown in Figure 4, CRR is different between rOMG and nOMG patients. At week 1, CRR was 18.18% (2/11) in the rOMG group and 37.5% (3/8) in the nOMG group. By the second week, CRR exceeded 50% in both groups. Ultimately, all patients achieved a 100% cumulative response by week 5, indicating a common onset of symptom improvement within 5 weeks.

**FIGURE 4:**
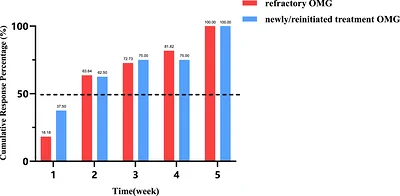
Cumulative response rates over time in refractory and newly/reinitiated treatment OMG patients. The refractory group (*n* = 11) and newly/reinitiated treatment group (*n* = 8) showed progressive increases in CRR from week 1 to week 5, reaching 100% in both groups by week 5. CRR was defined as the proportion of patients who had achieved a ≥1‐point reduction in the MG‐ADL score from baseline at or before each assessment.

#### 3.5.2 Prednisone Dosage Trajectories

LMM was also applied to evaluate longitudinal changes in daily prednisone dosage. Prednisone dosage showed a significant downward trend over time in both groups (Friedman test, *p* < 0.001 for both groups). The estimated mean monthly reduction was 2.28 mg in the nOMG group and 2.03 mg in the rOMG group; no statistically significant difference in prednisone‐dose trajectories was detected between the groups (time × group interaction *β* = 0.25, 95% CI: −1.26 to 1.77, *p* = 0.739).

As shown in Table 4, the median daily prednisone dose at the initial point was 30.00 mg (IQR 17.50–50.00) for the rOMG group, compared to 17.50 mg (IQR 15.00–22.50) for the nOMG group (p  = 0.335). By month 3, doses were substantially reduced in both groups to a comparable level: 5.00 mg (IQR 5.00–15.00) in the rOMG group, an 83.33% reduction from baseline, and 10.00 mg (IQR 6.25–12.50) in the nOMG group, a 42.86% reduction (p  = 0.590). At the final follow‐up, both groups maintained low‐dose regimens, with the rOMG group at 2.50 mg (IQR 2.50–5.00), a 91.67% reduction from baseline, and the nOMG group at 5.00 mg (IQR 3.75–7.50), a 71.43% reduction from baseline (p  = 0.251). All patients in both groups achieved substantial and clinically relevant reductions in prednisone use.

**TABLE 4:** Comparison of the median daily prednisone dosage at baseline, 3 months, and 6 months between the refractory and newly/reinitiated treatment groups.

Time	Refractory (IQR) (mg/day)	Newly/reinitiated treatment (IQR) (mg/day)	*Z* score	*p* value
Baseline	30.00 (17.50‐50.00)	17.50 (15.00‐22.50)	−0.96	0.335
3 months	5.00 (5.00‐15.00)	10.00 (6.25‐12.50)	−0.54	0.590
6 months	2.50 (2.50‐5.00)	5.00 (3.75‐7.50)	−1.15	0.251

Abbreviations: IQR, interquartile range; Z, Mann–Whitney test.

## 4 Discussion

In this multicenter real‐world cohort, telitacicept was associated with rapid and sustained clinical improvement in OMG, along with a progressive reduction in daily prednisone doses. By 6 months, 68.4% of the patients achieved MSE, and 84.2% were receiving ≤5 mg/day—a target widely recognized as the optimal long‐term maintenance dose in MG management (Imai et al. 2020; Narayanaswami et al. 2021). All patients demonstrated an initial clinical response within 5 weeks of treatment initiation. This is the first study to evaluate telitacicept in OMG stratified by prior treatment history. Notably, nOMG exhibited a trend toward faster early improvement; however, no statistically significant difference in outcomes was detected between the two groups. LMM analysis of the entire 6‐month trajectory detected no significant difference in the overall rate of improvement between the groups. These findings suggest that telitacicept‐based regimens may represent a promising treatment strategy for OMG, enabling concurrent corticosteroid reduction across the spectrum of prior treatment experience.

An early clinical response was observed following telitacicept treatment, with all 19 patients achieving an initial response within 5 weeks and 26.3% showing improvement as early as week 1. This trajectory aligns with the phase 2 GMG trial by Yin et al. (2024), which reported significant clinical improvements by week 4.

While direct cross‐study comparisons are limited by differences in patient populations and study designs, our observed 68.4% MSE rate aligns well with, or numerically surpasses, rates reported for conventional immunosuppressants (45.2%), eculizumab (21.4%), and efgartigimod (44.6%) in GMG (Bril et al. 2024; Vissing et al. 2020; Uzawa et al. 2023), as well as the 33.3% reported in a Bayesian study of telitacicept in GMG (Duan and Xiao 2026). This is the first study to evaluate MSE as a prespecified endpoint in OMG and to examine MSE rates stratified by prior treatment history. We also introduced “duration of sustained MSE” as a novel endpoint; among 13 MSE achievers, nine maintained MSE for >3 months, and two for up to 6 months. The observed sustained improvement may be biologically consistent with dual BAFF/APRIL blockade; however, the absence of immunological biomarker data precludes conclusions regarding the underlying mechanism (Yao et al. 2021; Vincent et al. 2013).

It is worth noting that all patients but one in our study adopted a low‐dose or moderate‐dose prednisone regimen combined with telitacicept. So, the rapid onset of our regimen should be interpreted as the effect of the combined immunomodulatory strategy, rather than a monotherapy effect of telitacicept. However, as widely documented in the literature, conventional low‐dose corticosteroids in OMG typically require 14 weeks to achieve clinical response (Benatar et al. 2016), which is significantly slower than the onset time observed in our study. It suggests that telitacicept may have played a significant role in the combined regimen.

Achieving minimal corticosteroid exposure is a key therapeutic goal. Prolonged steroid use drives metabolic and skeletal morbidity (Evoli and Damato 2023; Menon et al. 2021; Kaminski and Denk 2022). Our 75% prednisone reduction at 6 months, with 84.2% reaching ≤5 mg/day, aligns with the 76% reduction reported in a Bayesian GMG study (Duan and Xiao 2026), and the slow tapering achieved with traditional agents, which require 6–18 months (Farmakidis et al. 2018). Initial prednisone doses varied from 10 to 60 mg/day and were lower in the nOMG group than in the rOMG group (median 17.5 mg/day vs. 30.0 mg/day). Clinical improvement was observed in both groups; however, because prednisone dosing and tapering were not standardized, these findings cannot establish whether lower starting doses are sufficient when combined with telitacicept. This possibility warrants prospective evaluation.

Subgroup analysis showed final MSE proportions of 72.7% in the rOMG group and 62.5% in theChinese Society of Neuroimmunology. 2025. “Chinese Guideline for Diagnosis and Treatment of Myasthenia Gravis (2025 Edition).” Chinese Journal of Neurology 58, no. 7: 721–741. [In Chinese] nOMG group; no statistically significant difference in outcomes was detected between the two groups. LMM analysis of the entire 6‐month trajectory detected no significant difference in the overall rate of improvement. However, given our small sample size and baseline imbalances, this exploratory subgroup comparison is indeed statistically underpowered. Previous studies support biological heterogeneity among antibody‐producing cell populations and the role of BAFF/APRIL‐family signaling in B‐cell and plasma‐cell survival (Farmakidis et al. 2018; Croft 2009; Lazaridis and Tzartos 2020). Despite delayed ADL response, the refractory group showed aggressive early steroid tapering (83.3% by week 12), underscoring telitacicept's capacity (Wu et al. 2025).

Conceptually, telitacicept differs from downstream inhibitors (complement inhibitors, neonatal Fc receptor [FcRn] antagonists) that provide rapid but often non‐durable relief. Real‐world data show 35.9% of patients discontinued efgartigimod due to insufficient efficacy (Fuchs et al. 2026), and cyclical IgG fluctuations require individualized dosing (Habib et al. 2025; Konno et al. 2024). Evidence for FcRn antagonists in OMG remains sparse (Ma et al. 2024). Rituximab is limited by CD20‐negative long‐lived plasma cells that evade depletion (Menon and Bhandari 2025). Hypothetically, dual BAFF/APRIL inhibition by telitacicept may address this limitation by depriving these cells of key survival signals (Hiepe and Radbruch 2016), which could theoretically contribute to more sustained control. However, whether this proposed cellular mechanism accounts for the clinical observations in our cohort requires further validation with immunological biomarkers. Short‐term tolerability in this cohort was acceptable, consistent with pivotal trials (Yao et al. 2021; Yin et al. 2024), with only mild injection‐site reactions.

Limitations include small sample size, short follow‐up, and medication heterogeneity and baseline imbalances (particularly disease duration), potential underreporting of adverse events due to retrospective design, and LMM's assumption of linear trajectories. The lack of a control group precludes direct comparison with standard therapies. The concurrent initiation of prednisone and telitacicept in the newly/reinitiated treatment OMG group introduces substantial confounding. The subgroup comparisons are strictly exploratory generating. Despite this, targeted biologic development in OMG is advancing, with ongoing Phase 3 trials of efgartigimod (NCT06558279) and telitacicept (NCT07249632). Our study provides the first real‐world data on telitacicept in OMG stratified by prior treatment history, complementing these registration trials. Further research should involve designing a randomized controlled study, selecting patients (untreated or refractory), standardizing the treatment plan (such as whether to combine prednisone or not), and extending the observation period to assess risk of generalization.

In conclusion, in this small multicenter retrospective cohort, telitacicept‐based regimens were associated with improvement in MG‐ADL scores and reduced prednisone exposure over 6 months, with no serious adverse events documented. No statistically significant differences in clinical or prednisone‐dose trajectories were detected between the groups defined by prior treatment history. Prospective controlled studies are required to confirm these findings.

## Author Contributions

**Siyu Xie**: methodology, writing – original draft, data curation, software. **Wenli Song**: validation, formal analysis. **Ran Guo**: conceptualization, supervision. **Chunrong Li**: visualization, project administration. **Manman Zhang**: project administration. **Yifan Cheng**: visualization, software. **Wenting Guo**: validation. **Bo Jin**: funding acquisition, validation. **Jia Li**: supervision, methodology. **Yiqi Wang**: conceptualization, project administration, supervision, writing – review and editing.

## Funding

This work was supported by the National Natural Science Foundation of China (grant number: 82001366) and the Medical Science and Technology Project of Zhejiang Province (grant numbers 2022KY506 and 2024KY025).

## Ethics Statement

We confirm that we have read the journal's position on issues involved in ethical publication and affirm that this report is consistent with those guidelines.

## Conflicts of Interest

The authors declare no conflicts of interest.

## Supporting information




**Supplementary Material**: brb371816‐sup‐0001‐SuppMat.docx

## Data Availability

The data that support the findings of this study are available from the corresponding author upon reasonable request.
