# Supplementary material for: Real‐World Clinical Outcomes and Corticosteroid Tapering in Telitacicept‐Based Therapy for Ocular Myasthenia Gravis: An Exploratory Analysis by Prior Treatment History
Source: Brain Behav. 2026 Oct 8;16(10):e71816. doi: 10.1002/brb3.71816 (PMC13650419; doi:10.1002/brb3.71816)
Supplement: Supplementary file 1 [file BRB3-16-e71816-s001.docx]

Supplementary Table S1. Individual Telitacicept dosing regimens and interval adjustments over 6 months.

| **Patient No.** | Subgroup | Initial Dosing Regimen | Overall 6-Month Dosing and Interval Extension Trajectory |
| --- | --- | --- | --- |
| P1 | rOMG | 160 mg QW | 160 mg QW (Months 1–3),160 mg Q2W (Months 4–6) |
| P2 | rOMG | 160 mg QW | 160 mg QW (Months 1–3), 160 mg Q2W (Months 4–5), 160 mg Q3W (Month 6) |
| P3 | rOMG | 160 mg QW | 160 mg QW (Months 1–3), 160 mg Q2W (Months 4–5), 160 mg Q3W (Month 6) |
| P4 | rOMG | 240mg QW | 240 mg QW (Months 1–3), 240 mg Q2W (Months 4–5), 240mg Q3W (Month 6) |
| P5 | rOMG | 240mg QW | 240 mg QW (Months 1–3), 240 mg Q2W (Months 4–5), 240mg Q3W (Month 6) |
| P6 | rOMG | 160 mg QW | 160 mg QW (Months 1–3), 160 mg Q2W (Months 4–5), 160 mg Q3W (Month 6) |
| P7 | rOMG | 240mg QW | 240 mg QW (Months 1–3), 240 mg Q2W (Months 4–5), 240mg Q3W (Month 6) |
| P8 | rOMG | 160 mg Q2W | 160 mg Q2W (Months 1–4), 80 mg Q2W (Months 5–6) |
| P9 | rOMG | 160 mg QW | 160 mg QW (Months 1–3), discontinued after Month 3 |
| P10 | rOMG | 160 mg QW | 160 mg QW (Months 1- 5),160 mg Q2W (Month 6) |
| P11 | rOMG | 160 mg Q2W | 160 mg Q2W maintained for 6 months |
| P12(N1) | nOMG | 160 mg QW | 160 mg QW (Months 1- 5),160 mg Q2W (Month 6) |
| P13(N2) | nOMG | 160 mg QW | 160 mg QW (Months 1–2),160 mg Q2W (Months 3–6) |
| P14(N3) | nOMG | 160 mg QW | 160 mg QW (Month 1),240 mg QW (Month 2),160 mg QW (Months 3–4),160 mg Q2W (Months 5–6) |
| P15(N4) | nOMG | 160 mg QW | 160 mg QW maintained for 6 months |
| P16(N5) | nOMG | 240mg QW | 240 mg QW (Months 1–3), 240 mg Q2W (Months 4–5), 240mg Q3W (Month 6) |
| P17(N6) | nOMG | 160 mg QW | 160 mg QW (Months 1–2),160 mg Q2W (Months 3–6) |
| P18(N7) | nOMG | 160 mg QW | 160 mg QW maintained for 6 months |
| P19(N8) | nOMG | 240mg QW | 240 mg QW (Months 1–3), 240 mg Q2W (Months 4–5), 240mg Q3W (Month 6) |

rOMG, refractory Ocular Myasthenia Gravis; nOMG, newly/reinitiated treatment Ocular Myasthenia Gravis; Q1W, once weekly; Q2W, once every 2 weeks; Q3W, once every 3 weeks.

Supplementary Table S2. Summary of Treatment-Emergent Adverse Events (TEAEs) Over 6 months.

| Adverse Event Type | Patients, n (%) | CTCAE v5.0 Grade | Onset Time | Action Taken | Outcome |
| --- | --- | --- | --- | --- | --- |
| Serious Adverse Events (SAEs) | 0 (0.0%) | N/A | N/A | None | N/A |
| Discontinuation due to AEs | 0 (0.0%) | N/A | N/A | None | N/A |
| Injection-site reactions | 3 (15.79%) | Grade 1 (Mild) | Weeks 1–4 | None (Self-limiting) | Fully resolved |
| Injection-site erythema | 3 (15.79%) | Grade 1 (Mild) | Weeks 1–4 | None | Resolved |
| Injection-site mild pain | 1 (5.26%) | Grade 1 (Mild) | Week 1 | None | Resolved |
| Infections (e.g., upper respiratory) | 0 (0.0%) | N/A | N/A | None | N/A |

TEAEs, treatment-emergent adverse events; CTCAE, Common Terminology Criteria for Adverse Events (version 5.0).
